# Screening for Brain Metastases in Patients With NSCLC: A Qualitative Study on the Psychologic Impact of Being Diagnosed With Asymptomatic Brain Metastases

**DOI:** 10.1016/j.jtocrr.2022.100401

**Published:** 2022-08-27

**Authors:** Janna J.A. O. Schoenmaekers, Jeroen Bruinsma, Claire Wolfs, Lidia Barberio, Anita Brouns, Anne-Marie C. Dingemans, Lizza E.L. Hendriks

**Affiliations:** a Department of Pulmonary Diseases, GROW—School for Oncology and Reproduction, Maastricht University Medical Center+, Maastricht, The Netherlands; b Department of Health Promotion, Maastricht University, Maastricht, The Netherlands; c Department of Psychiatry and Neuropsychology, Maastricht University Medical Center+, Maastricht, The Netherlands; d Longkanker Nederland, Utrecht, The Netherlands; e Department of Pulmonary Diseases, Zuyderland Medical Center, Heerlen, The Netherlands; f Department of Pulmonary Diseases, Erasmus MC Cancer Institute, University Medical Center, Rotterdam, The Netherlands

**Keywords:** Qualitative study, NSCLC, Brain metastases, Distress, Scanxiety

## Abstract

**Introduction:**

The brain is a frequent site of metastases in NSCLC, and screening for asymptomatic brain metastases (BM) is increasingly advised in NSCLC guidelines. An asymptomatic BM diagnosis may trigger anxiety for future neurologic problems and can negatively affect quality of life of patients and their relatives. Therefore, we performed this qualitative study.

**Methods:**

Three focus group discussions were organized with patients with NSCLC and asymptomatic BM (N = 3–4 per group) and separately with their relatives, to explore this psychosocial impact. Two researchers independently performed an inductive content analysis.

**Results:**

A total of 10 patients and 10 relatives participated in six focus groups. A diagnosis of BM caused feelings of distress and anxiety in both patients and relatives. These feelings diminished over time in case of a tumor responding to systemic therapy. The diagnosis of BM was not perceived as more distressful than other metastases, and scan-related anxiety was not experienced. Although magnetic resonance imaging screening and follow-up were thought of as burdensome, follow-up was valued. The coping strategies of both groups seemed related to personality and to the efficacy of the given systemic therapy. Relatives appreciated peer support of other relatives during the focus groups, and they seemed open for future psychological support.

**Conclusions:**

Asymptomatic BM diagnosis can cause anxiety and distress, but this diminishes over time with effective systemic treatment. Although patients perceive magnetic resonance imaging as burdensome, they value follow-up screening and imaging. Relatives highly appreciated peer support, and psychological distress of relatives should not be overlooked.

## Introduction

Brain metastases (BM) are diagnosed in 40% to 70% of patients with metastatic NSCLC, during the course of the disease.^1^, ^2^, ^3^ Historically, BM were associated with poor overall survival and low quality of life (QoL).^4^^,^^5^ Nevertheless, owing to the introduction of targeted therapies for patients with an oncogenic driver and immune checkpoint inhibitors for most of the other patients, the overall survival probability of patients with NSCLC is increasing.^1^^,^^6^

Throughout almost all disease stages, the screening for asymptomatic BM is advised in NSCLC guidelines, except for patients in a (very) early disease stage as the risk of BM is low in these stages.^7^, ^8^, ^9^ The two main reasons for screening are to adjust therapy if asymptomatic BM are detected in those with extracranial localized disease or to select the optimal treatment strategy in those with metastasized disease. In the former, this implies either oligometastatic treatment or switch from radical-intent therapy to palliative therapy. In the latter, a choice has to be made between upfront local BM treatment and systemic therapy followed by local treatment on (symptomatic) BM progression.^10^ Furthermore, regular brain imaging during follow-up is advised in patients at high risk of BM, to have a possibility to treat BM before they become symptomatic.^9^^,^^11^

Previous studies revealed that a diagnosis of BM, subsequently needing treatment with radiotherapy, has a profound impact on patients and may cause feelings of anxiety or fear of dying, loss of control, impaired cognition, or changes in personality.^12^ Similar results were found for patients with a primary brain tumor diagnosis and their spouses.^13^ Nevertheless, it is not yet known whether this is also the case in asymptomatic BM. It has been found that follow-up scans (not specifically evaluated for brain imaging) may also have a negative impact on the QoL as these can induce feelings of scan-associated anxiety (scanxiety).^14^^,^^15^ It is not known whether this also accounts for the follow-up with brain imaging.

Eggen et al.^16^ report that anxiety for dying in patients with NSCLC and BM (N = 78, 53% with BM) is common but not necessarily attributed to the BM diagnosis. Nevertheless, the patients included in this study were diagnosed with symptomatic BM making it difficult to extrapolate the results to patients with asymptomatic BM. Currently, there is only limited understanding in how asymptomatic BM affect the psychological well-being and perceived QoL of patients and their relatives. More insight helps to obtain directions to optimize postdiagnostic psychological support and shared decision making with patients for whom there are conflicting data whether BM screening is necessary. Therefore, this qualitative study used focus group discussions to investigate the perspective of patients. In addition, the perspectives of their family members or close friends were explored.

## Materials and Methods

Focus group discussions were organized with persons having NSCLC with asymptomatic BM. Separate focus groups were also organized, including one family member or close friend of each patient. These focus groups allowed for an in-depth understanding in the experiences throughout the disease trajectory and the psychological impact of having NSCLC with asymptomatic BM. The findings are reported according to the consolidated criteria for reporting qualitative research.^17^

### Participants

Participants were enrolled from the thoracic oncology outpatient clinic of the comprehensive cancer center at Maastricht University Medical Center, The Netherlands. They were selected and approached by the treating physician. When participants where interested, the information was given to the researcher, the researcher provided the patient information form to the participants several days before the focus group session, and all participants signed informed consent at the start of the focus group session. Participants were eligible if diagnosed with having NSCLC and asymptomatic BM, irrespective of timing of the diagnosis. Eligibility was regardless of the presence of an oncogenic driver or current systemic treatment. Patients were excluded if they were unable to interact in focus groups, for example, because of a neurologic illness. One family member or a close friend (who were highly involved) of each patient was also invited for a separate focus group.

### Focus Groups

The focus groups were conducted by four experienced and trained researchers (JS: pulmonologist; female; CW: neuropsychologist, female; AB: pulmonologist, female; JB: PhD in neuropsychology, male). Each focus group was moderated by a researcher (CW or JB) who had experience with qualitative research and (neuro)psychology. The two other researchers had clinical experience in thoracic oncology and acted as an assistant moderator and scribe (JS and AB). It was possible that the participants knew the assistant moderators; however, the researchers who conducted the interviews were not known by the participants. Each focus group discussion included three/four participants. In total, six focus groups were organized lasting 1.5 hours (i.e., three with patients with NSCLC and three with family and friends). In case no data saturation was established after these focus group sessions, an extra focus group would be organized.

The focus group was held in a meeting room in the hospital, in a quiet setting. Each focus group started with a 15-minute welcome in which participants completed a short questionnaire on sociodemographics (i.e., marital status, having children, and highest received education). Subsequently, predefined questions were asked on the feelings (fears and anxiety) about having BM, the impact of having BM versus other metastases, experience with imaging and scanxiety, and the use of coping strategies. An English translation of the interview questions is provided as Supplementary Material 1.

### Analysis

The focus group discussions were audiorecorded and transcribed verbatim. The first and second authors independently performed a content analysis by openly coding the data using ATLAS.ti version 9. Through the discussion, they reached consensus about the used codes and defined important (sub)categories from the data. Subsequently, these (sub)categories were visualized, and the main findings were discussed within the research team including a neuropsychologist, a health scientist, and experts in thoracic oncology.

### Ethical Consideration

This study was approved by the Medical Ethics Committee of the Maastricht University Medical Center (METC2017-0286). All participants provided written informed consent before participating in the focus groups.

The study is conducted according to the principles of the Declaration of Helsinki (Brazil 2013)^18^ and in accordance with the Medical Research Involving Human Subjects Act (WMO).

## Results

### Participants

Between May 2018 and July 2020, a total of 21 patients having NSCLC with asymptomatic BM were approached for participation. Eventually, 13 patients were willing to participate and were included in the focus groups. The other approached patients (n = 8) refused participation because they did not feel the need to talk about the diagnosis (n = 5); the partner was not willing to participate (n = 1); or they felt that extra hospital visits were burdensome (n = 2). In separate focus groups, one family member or close friend of each patient participated.

Eventually, one focus group could not be analyzed owing to technical problems with the audiorecorder. Therefore, a fourth round of focus groups was organized. As a result, the analysis includes data from 10 patients and 10 family members or close friends. After these focus group sessions, data saturation was reached and another round was not necessary.

Many of the patients were of female sex (60%), and the mean age was 66 years (range: 57–75 y) (Table 1). In addition, most was married (80%) and 50% had children. Most patients (90%) had an adenocarcinoma of which 90% had an oncogenic driver. The median time between the first diagnosis of NSCLC and the diagnosis of asymptomatic BM was 32.3 months, ranging from 0 to 64.6 months. The median time between the diagnosis of asymptomatic BM and participation in the focus groups was 21.6 months, ranging from 2.5 to 40.7 months. Among the participating relatives, 80% was the partner of the patient and 20% was a close friend (Table 2).

**Table 1:** Characteristics of the Patients

Baseline Characteristics	n (%) Total N = 10
Sex	
Female	6 (60)
Male	4 (40)
Age in y (range)	66.1 (57–75)
Marital status	
Living together	0 (0)
Married	8 (80)
Single	2 (20)
Patients having children	
Yes	5 (50)
No	5 (50)
Highest education received	
Primary education	1 (10)
Secondary education	3 (30)
College	2 (20)
University of applied sciences	3 (30)
University	1 (10)
Disease diagnosis	
NSCLC (adenocarcinoma)	9 (90)
NSCLC (NOS)	1 (10)
Median time in mo between diagnosis NSCLC and BM (range)	32.3 (0–64.6)
Median time in mo between diagnosis of BM and the focus group (range)	21.6 (2.5–40.7)
Mutational status	
EGFR mutation	5 (50)
Exon 19 deletion	1 (10)
Exon 20 insertion	1 (10)
Exon 21	3 (30)
ALK translocation	3 (30)
BRAF V600E	1 (10)
No oncogenic driver	1 (10)
Stage (TNM eighth) at first diagnosis	
Stage III	3 (30)
Stage IV	7 (70)
Imaging modality used to diagnose BM	
MRI	9 (90)
CT	1 (10)
BM discovered on first cerebral imaging	
Yes	6 (60)
No, in follow-up	4 (40)
Treatment at the moment of the focus group	
Targeted therapy	8 (80)
Chemoradiotherapy (oligometastatic treatment)	2 (20)
Treated with local BM-directed therapy	
Yes	4 (40)
No	6 (60)

BM, brain metastases; CT, computed tomography; MRI, magnetic resonance imaging; NOS, not otherwise specified.

**Table 2:** Characteristics of the Family and Friends

Characteristics of the Family and Friends	N = 10, n (%)
Gender	
Female	6 (60)
Male	4 (40)
Age in y (range)	65 (56–73)
Relationship with patient	
Partner	8 (80)
Close friend	2 (20)
Highest education received	
Primary education	0 (0)
Secondary education	3 (30)
College	2 (20)
University of applied sciences	4 (40)
University	1 (10)

### Analysis of the Focus Groups

Four major themes concerning the psychosocial impact of having asymptomatic BM in patients with NSCLC were identified, and these are discussed subsequently.

#### Coping With the Diagnosis of BM

According to the patients, the diagnosis of NSCLC had a profound impact and caused feelings of distress and anxiety. Some patients felt that the accompanying diagnosis of BM intensified the experienced anxiety.

“I was shocked by the diagnosis of BM because I didn’t feel anything.” Female, age 70 years, *EGFR* mutation

Strategies used by patients to cope with the diagnosis of BM were linked to their personality. Some patients described that their glass is always half-full, whereas others felt it was half-empty. This influenced how they coped with feelings of distress in daily life.

“I try to live in the moment and plan fun activities as much as possible. Sometimes I even take advantage of my disease. For example, when there is a concert and I can get a better seat because of my disease.” Female, age 59 years, *ALK* translocation

“I simply can’t accept the diagnosis and can’t deal with it. (…). I worry about it a lot, especially at night. I’m afraid for what the future will bring.” Male, age 74 years, *EGFR* mutation

There was a wide variety of coping strategies used by the patients, their need for information, and preferences regarding shared decision making. Some placed all their trust in their treating physician and did not want to discuss treatment options (i.e., they did not prefer shared decision making). Others searched for detailed information on the internet and expressed that they liked to ask a lot of questions during medical consultation.

“I want a short consult and do not have many questions.” Male, age 66 years, *EGFR* mutation

“When I go to the doctor’s office, often with my whole family, it is an active consult where we address many of our questions.” Female, age 61 years, *EGFR* mutation

Anxiety caused by having BM seemed related to the time between the diagnosis of BM and participation in the focus groups. More specifically, patients with a short interval experienced more feelings of distress and anxiety compared with patients who had a longer interval between the diagnosis of BM and effective treatment and participation in the focus groups.

“In the beginning I experienced anxiety about the possible symptoms that may develop due to the BM. Later, these feelings changed and I felt less worried because I can still do everything.” Female, age 70 years, *EGFR* mutation

Coping of the participating relatives also depended on their personality and need for information. The participating partners and close friends experienced difficulty with coping owing to the uncertainty that accompanied the NSCLC and BM prognosis. For example, they experienced that this had changed their future perspective and limited their ability to make plans for the future. Some described that the best strategy to cope with the uncertainty was to live by the day.

“It feels like were are living with a time bomb.” Female, age 63 years, *EGFR* mutation

#### Impact of Imaging to Screen for and to Follow Up BM

All patients expressed a strong preference for regular imaging to screen for new or progressing BM, even if this could lead to the discovery of new BM, whereas local treatment of these BM is not always necessary. They experienced that not knowing about new BM is worse.

“Even though the local therapy did not start immediately and I did not have any symptoms of the discovered BM, I would still want to know that I have BM.” Female, age 72 years, *BRAF* V600E

All patients considered imaging of the brain with a magnetic resonance imaging (MRI) scan as burdensome. They felt that these scans take a long time to be performed while they need to lie very still. Sometimes, this led to physical complaints, such as a back pain. Despite the burden, most patients perceived that it is worth it because an MRI is more sensitive in diagnosing BM compared with a computed tomography (CT) scan. Only one patient felt that it is less important to diagnose the small BM.

“Just lie down, close your eyes and focus on something else or think about fun stuff.” Female, age 70 years, *ALK* translocation

The patients did not express a preference about the interval of the follow-up imaging of the brain.

“How often and when imaging should be done has to be decided by the physician.” Male, age 75 years, *ALK* translocation

#### Difference Between Having BM Versus Having Metastases at Other Sites

Most patients did not feel more distressed about BM compared with having other metastases. The experienced level of distress was linked to having relatives with specific types of cancer (including sites of metastases causing complaints) or other neurologic problems.

“My niece died at a young age due to a brain tumor. So I was not that happy when I heard about the diagnosis of BM.” Female, age 63 years, *EGFR* mutation

“I’m afraid of developing dementia. My neighbor had dementia and this is a doomsday scenario.” Male, age 75 years, *ALK* translocation

Some patients experienced more fear for disabling constraints of symptoms caused by other metastases. For example, one patient was more concerned of becoming paralyzed owing to bone lesions than feeling anxious for possible cognitive decline. Other participants also felt more afraid of symptomatic metastases in other places, such as bone metastases.

“I’m afraid of bone metastases in the vertebra because you can get paralyzed. I’m certainly not waiting for that.” Female, age 63 years, *EGFR* mutation

#### Involvement of Family and Friends

According to most patients, they had informed family and friends about the NSCLC diagnosis and the BM. Mostly, family and friends had not responded differently to the NSCLC or BM diagnosis. Especially, the first time after the diagnosis, family and friends reacted shocked and were supportive. According to most participants, the high level of involvement and support diminished over time.

“We told everybody about the diagnosis. In the first months family and friends were very involved. However, over time their involvement diminished.” Male, age 66 years, *EGFR* mutation

The coronavirus disease 2019 (COVID-19) pandemic had a profound impact on the involvement of family and friends. The last focus group was organized during the pandemic (after it was allowed according to the local COVID-19 measures). The patients told that they had isolated themselves because they feared getting COVID-19. Although they kept in contact with family and friends by means of telephone or digitally, some experienced feelings of loneliness.

“I experience a lot of support from close family and would receive daily text messages. However, I deliberately keep social distance to protect myself for COVID-19. At times, this is very lonely.” Male, age 74 years, *EGFR* mutation

The participating family and close friends reported this as well. They told that giving and receiving support was challenging during the COVID-19 pandemic. They also experienced it as difficult and impersonal to bring bad news by means of the phone or digitally. They missed the real-life and physical contact with family and friends.

After the focus groups, some family and friends clearly expressed to highly appreciate having contact with peers during the focus groups. This was especially attributed to the feeling that they could openly talk about their experiences without having to weigh every word because the patients were not present. They also felt that there is only limited recognition for the psychological impact of lung cancer and BM on the involved relatives and friends.

## Discussion

The results of this study reveal that a diagnosis of asymptomatic BM leads to feelings of distress and anxiety in patients and close relatives and friends, especially, in an early stage after the diagnosis. Most patients did not perceive the diagnosis of BM as more distressful compared with the diagnosis of other metastases. Although most patients experienced MRI as burdensome, most preferred MRI over CT scan. They attributed this to the sensitivity of the MRI in detecting BM. Patients did not express a preference for the timing of follow-up imaging and felt that it was up to the oncologist to decide. Our findings also indicate that patients and their close family or friends can use a wide variety of coping strategies and have diverse needs for information and shared decision making. This appeared to be related to their personality but also to the stage in the disease process. At first, family and friends often reacted shocked about the diagnosis of lung cancer and BM and were very supportive. Nevertheless, according to most participants, their high level of involvement and support diminished over time. The close relatives who participated in the focus groups experienced that there was only limited psychological support for themselves.

Anxiety and distress are common in patients with metastatic cancer. Nevertheless, as far as we know, there is no knowledge about the impact of asymptomatic BM in patients with NSCLC on levels of anxiety and distress. A previous study by Eggen et al.^16^ (a cross-sectional pilot study with questionnaires and neuropsychological testing) revealed that death anxiety was reported in 43% of the participating patients with metastasized NSCLC (n = 78, 53% BM, all symptomatic). These levels of anxiety did not differ between the patients with and without BM; however, these were all patients with BM-related symptoms.^16^ Furthermore, a study by Cordes et al.^12^ using the validated National Comprehensive Cancer Network Distress Thermometer and the Hospital Anxiety and Depression Scale, comparing patients with BM from different solid tumors treated by radiotherapy, with patients with breast cancer without cranial involvement receiving adjuvant whole breast radiotherapy, revealed that the course of distress, anxiety, and depression did not differ significantly between those groups (*p* = 0.029). Our study reveals that the experienced levels of distress caused by BM were particularly evident after receiving the BM diagnosis but diminished over time, especially after nonprogressive disease owing to effective systemic therapy.

Our study also provides unique insight in “scanxiety” and burden experienced by patients with metastatic NSCLC. Previous research revealed that approximately half of the patients with advanced cancer (n = 222) experience feelings of scanxiety.^19^ Our findings confirm that an MRI is experienced as more burdensome compared with a CT or positron emission tomography-CT scan because an MRI is accompanied by psychological and physical side effects (such as claustrophobia or scan-related noise) that adversely affect the scan experience.^20^ Nevertheless, similar to earlier findings of Bui et al.,^21^ (a qualitative study with semistructured interviews; 16 participants) our study reveals that most patients accepted this as part of the treatment trajectory. Most patients felt that especially waiting for the results of the scan was stressful. Our study also reveals that despite the burden most patients preferred an MRI because it is more sensitive in detecting BM compared with other imaging techniques.^22^

This study also allows insight in the psychological impact of BM versus metastases at other sites. Most patients did not experience more feelings of anxiety or distress owing to the BM. In contrary, some patients felt that the disabling consequences of other metastases were more frightening than the possibility of cognitive decline caused by BM. These findings correspond with earlier research of Ecclestone et al.,^23^ who revealed that patients with breast cancer and BM experienced a higher QoL versus patients with bone metastases (n = 174, 12 patients with BM).

A unique element of this study is that it also allowed an exploration of the experiences and perspectives of close family and friends of patients with BM. These family and friends appreciated the focus groups and experienced it as a form of peer support contact. They deliberately stated to miss professional psychological support during the course of the disease. It is known that providing informal care to patients with cancer can be stressful, demanding, and burdensome.^24^ Providing informal care can inflict emotional, social, physical, financial, and spiritual strain on the involved relatives.^25^ The well-being of family and friends is important because stress in informal caregivers may also cause higher levels of anxiety and depressive symptoms in patients.^26^ Therefore, the identification and management of caregiver burden are important considerations for a comprehensive cancer care program.

Focus groups are frequently used in health research and allow a detailed understanding in the experiences of the participants by means of active discussion.^27^^,^^28^ The method promotes self-disclosure by enhancing group interaction, and this can help participants in sharing their views and attitudes.^28^ A possible disadvantage of focus group methodology is that participants may provide socially desirable answers instead of expressing an honest opinion. For example, participants could hesitate to express thoughts that oppose the views of other participants. One-on-one interviews could overcome this limitation and could have aided to further deepen the findings of this study. A second limitation is that most of the included patients had an oncogenic driver mutation and were receiving systemic treatment with a tyrosine kinase inhibitor. Overall, these patients have a better prognosis than patients with stage IV NSCLC without a driver mutation.^29^^,^^30^ This may have biased our findings as our results reveal that nonprogressive patients experienced less anxiety and distress. These results could have been different for patients without an oncogenic driver. Nevertheless, as incidence of BM is high in patients with an oncogenic driver, we think that our results are important.^1^ Furthermore, screening in the follow-up is often advised for this subgroup of patients, adding even more value to our results.^11^

A third limitation includes the impact of the COVID-19 pandemic. During local COVID-19 restrictions, the last focus group was organized later than anticipated. The advantage is that it provided us with the opportunity to explore how patients experienced that period, although this was not an aim of the study.

Patients with NSCLC with asymptomatic BM experience high levels of anxiety and distress after receiving a BM diagnosis. The experienced levels of anxiety and distress diminish over time, especially when they have a nonprogressive disease trajectory owing to effective systemic treatment. Although most patients experience follow-up screening by MRI as burdensome, they accept this as part of the treatment trajectory and even prefer the MRI because of its better sensitivity. Another important conclusion is that the participating family and friends experienced the focus group as a psychosocial support, which is often lacking in daily care. In conclusion, from a patient perspective, the BM screening advice in clinical guidelines is supported, but in future clinical practice, more attention should be paid to psychological support, also for close relatives.

## CRediT Authorship Contribution Statement

**Janna J. A. O. Schoenmaekers:** Conceptualization, Methodology, Data curation, Formal analysis, Writing—original draft, Writing—review and editing.

**Jeroen Bruinsma:** Methodology, Data curation, Formal analysis, Writing—original draft, Writing—review and editing.

**Claire Wolfs:** Conceptualization, Data curation, Writing—review and editing.

**Anita Brouns:** Data curation, Writing—review and editing.

**Lidia Barberio:** Writing—review and editing.

**Anne-Marie C. Dingemans:** Conceptualization, Supervision, Writing—review and editing.

**Lizza E. L. Hendriks:** Conceptualization, Funding acquisition, Supervision, Writing—review and editing.
