# Supplementary material for: Screening for Brain Metastases in Patients With NSCLC: A Qualitative Study on the Psychologic Impact of Being Diagnosed With Asymptomatic Brain Metastases
Source: JTO Clin Res Rep. 2022 Aug 27;3(10):100401. doi: 10.1016/j.jtocrr.2022.100401 (PMC9516448; doi:10.1016/j.jtocrr.2022.100401)
Supplement: Supplementary Material 1 [file mmc1.docx]

Supplemental material S1: Guiding questions focus group

- Introducing moderators and focus group participants
- How would you describe yourself as a person (e.g. assertive or more of a wait and see attitude?)
- How did you feel when you were diagnosed with BM? Could you describe your feelings of that moment?
- Did you experience anxiety? And what is it that you are afraid of?
- Did you experience differences of emotions with hearing that you had lung cancer in comparison with the diagnosis of BM?
- Have you told your family and friends about the diagnosis of BM? And if you did, how did they respond?
- Do you feel like your familly and friends treat you differently since they know of the diagnosis BM (different than with the diagnosis lung cancer alone?)
- Did your feelings or concerns change when you were aware of the diagnosis BM for a longer period of time?
- Do you also have metastases on other sides?
- Is the feeling of having mestastases on other sides different in comparison with having BM?
- Are you well informed about (the consequences) of having BM?
- If you had the choice would you rather not know about the BM diagnosis?
- Is there any follow up of the BM with imaging? How often? If you had a choice would you rather not have follow up with imaging? What would be the best follow up frequency?
- What do you think about the scans? How do you feel the days before scanning? After a few follow up scans, does the feeling change?
- The best follow up imaging for the brain is a MRI, what do you think about that?
- If you had a choice, would you rather be followed up by MRI or CT?
- A part of the patients that have asymptomatic BM will not be treated locally, but first the effect of the systemic therapy will be awaited (chemotherapy, targeted therapy or immunotherapy). Is it a difference for you whether you will be treated with local or systemic therapy?
- Is your life changed since the knowledge of having BM?
- Are you afraid of the possible complaints you could get , eventhough you do not have complaints of the BM at the moment?
- Do you have a good contact with your family physician?
- What are your needs concerning the contacts with the hospital?
